# Achieving Optimal Medical Therapy: Insights From the ORBITA Trial

**DOI:** 10.1161/JAHA.120.017381

**Published:** 2021-01-26

**Authors:** Michael Foley, Christopher A. Rajkumar, Matthew Shun‐Shin, Sashiananthan Ganesananthan, Henry Seligman, James Howard, Alexandra N. Nowbar, Thomas R. Keeble, John R. Davies, Kare H. Tang, Robert Gerber, Peter O’Kane, Andrew S. P. Sharp, Ricardo Petraco, Iqbal S. Malik, Sukhjinder Nijjer, Sayan Sen, Darrel P. Francis, Rasha Al‐Lamee

**Affiliations:** ^1^ National Heart and Lung Institute Imperial College London London UK; ^2^ Imperial College Healthcare NHS Trust London UK; ^3^ Essex Cardiothoracic Centre Basildon UK; ^4^ Anglia Ruskin School of Medicine Chelmsford UK; ^5^ East Sussex Healthcare NHS Trust Hastings UK; ^6^ Royal Bournemouth and Christchurch NHS Trust Bournemouth UK; ^7^ University Hospital of Wales Cardiff UK

**Keywords:** adverse effects, angina, compliance/adherence, medical therapy, randomized controlled trial, Percutaneous Coronary Intervention, Compliance/Adherence, Cardiovascular Disease, Chronic Ischemic Heart Disease, Pharmacology

## Abstract

**Background:**

In stable coronary artery disease, medications are used for 2 purposes: cardiovascular risk reduction and symptom improvement. In clinical trials and clinical practice, medication use is often not optimal. The ORBITA (Objective Randomised Blinded Investigation With Optimal Medical Therapy of Angioplasty in Stable Angina) trial was the first placebo‐controlled trial of percutaneous coronary intervention. A key component of the ORBITA trial design was the inclusion of a medical optimization phase, aimed at ensuring that all patients were treated with guideline‐directed truly optimal medical therapy. In this study, we report the medical therapy that was achieved.

**Methods and Results:**

After enrollment into the ORBITA trial, all 200 patients entered a 6‐week period of intensive medical therapy optimization, with initiation and uptitration of risk reduction and antianginal therapy. At the prerandomization stage, the median number of antianginals established was 3 (interquartile range, 2–4). A total of 195 patients (97.5%) reached the prespecified target of ≥2 antianginals; 136 (68.0%) did not stop any antianginals because of adverse effects, and the median number of antianginals stopped for adverse effects per patient was 0 (interquartile range, 0–1). Amlodipine and bisoprolol were well tolerated (stopped for adverse effects in 4/175 [2.3%] and 9/167 [5.4%], respectively). Ranolazine and ivabradine were also well tolerated (stopped for adverse effects in 1/20 [5.0%] and 1/18 [5.6%], respectively). Isosorbide mononitrate and nicorandil were stopped for adverse effects in 36 of 172 (20.9%) and 32 of 141 (22.7%) of patients, respectively. Statins were well tolerated and taken by 191 of 200 (95.5%) patients.

**Conclusions:**

In the 12‐week ORBITA trial period, medical therapy was successfully optimized and well tolerated, with few drug adverse effects leading to therapy cessation. Truly optimal medical therapy can be achieved in clinical trials, and translating this into longer‐term clinical practice should be a focus of future study.

**Registration:**

URL: https://www.clinicaltrials.gov; Unique identifier: NCT02062593.

**Nonstandard Abbreviations and Acronyms**
ACME: Angioplasty Compared to Medicine
COURAGE: Clinical Outcomes Utilizing Revascularization and Aggressive Drug Evaluation
FAME 2: Fractional Flow Reserve Versus Angiography for Multivessel Evaluation 2
ISCHEMIA: International Study of Comparative Health Effectiveness With Medical and Invasive Approaches
ORBITA: Objective Randomised Blinded Investigation With Optimal Medical Therapy of Angioplasty in Stable Angina

**Clinical Perspective**

**What Is New?**

- In the ORBITA (Objective Randomised Blinded Investigation With Optimal Medical Therapy of Angioplasty in Stable Angina) trial, the first placebo‐controlled trial of percutaneous coronary intervention in stable angina, patients underwent a 6‐week medical optimization period before randomization to percutaneous coronary intervention or placebo.
- We demonstrate that in a clinical trial setting, truly optimal medical therapy can be successfully established and well tolerated, with 97.5% of patients taking ≥2 antianginal medications at 6 weeks.

**What Are the Clinical Implications?**

- It is possible to attain truly optimum medical therapy within trials of stable angina. This level of therapy may also translate into clinical practice.
- The impact of antianginal optimization on the placebo‐controlled effect of percutaneous coronary intervention is not known; this should be the subject of future research.

The treatment aims for patients with stable coronary artery disease (CAD) are to reduce the risk of future cardiovascular events and to improve angina symptoms.^1^ Reducing the risk of future cardiovascular events is achieved by slowing disease progression, reducing plaque instability, and reducing thrombus aggregation. This is achieved with lifestyle modification, high‐intensity statin therapy, and antiplatelets.^2^ Large randomized controlled trials in stable CAD have demonstrated that percutaneous coronary intervention (PCI) does not confer a survival benefit or a reduced risk of myocardial infarction, when added to optimal medical therapy.^3^, ^4^ In the ISCHEMIA (International Study of Comparative Health Effectiveness With Medical and Invasive Approaches) trial, even in the context of moderate or severe ischemia, PCI did not prevent myocardial infarction or death.^5^

The second aim of treatment is symptom relief. National and international guidelines recommend initial use of antianginal medications, which can be used in combination to reduce symptoms. If a patient’s angina is not well controlled with the use of short‐acting nitrates alone, recommended first‐line therapy is either a β blocker or a calcium channel antagonist. Second‐ and third‐line agents, including long‐acting nitrates, nicorandil, ivabradine, and ranolazine, should be chosen according to comorbidities, contraindications, patient preference, and medication cost. Revascularization is considered for patients who remain symptomatic despite treatment with at least 2 antianginal agents or are intolerant of medical therapy.^6^, ^7^

Initiating and uptitrating antianginal therapy can present a challenge in clinical practice for a variety of reasons. Engaging in an intensive process of antianginal medication introduction and uptitration may be seen as more time‐consuming than an upfront PCI procedure. Clinician and patient concern about polypharmacy can mean that antianginals are not introduced or uptitrated. Adverse effects may result in poor medication persistence and adherence, leading to patients not consistently taking enough antianginal therapy to effectively treat their symptoms.^8^ Medical therapy may also not be optimized because of discordance between clinician and patient estimation of angina.^9^

In the ORBITA (Objective Randomised Blinded Investigation With Optimal Medical Therapy of Angioplasty in Stable Angina) trial, patients with angina and single‐vessel severe coronary stenosis underwent a prespecified period of antianginal therapy uptitration. The medication optimization phase was intensive to achieve good levels of medical therapy in only 6 weeks.^10^ In this article, we describe the medical therapy that was achieved during the medical optimization and follow‐up phases. We report which drugs were best tolerated, the adverse effects experienced, and the low‐density lipoprotein and blood pressure achieved.

## Methods

The data, analytical methods, and study materials will not be made available to other researchers for the purposes of reproducing the results or replicating the procedure. The London Central Research Ethics Committee (reference 13/LO/1340) approved the study, and written consent was obtained from all patients before enrollment.

### Study Design

The methods of the ORBITA trial have been reported previously.^10^ In brief, patients were eligible for recruitment if they had symptoms of angina and ≥1 significant angiographic stenosis (≥70%) in a single coronary artery. Medical therapy and symptoms at enrollment were recorded. Study participants subsequently entered a 6‐week medical optimization phase.

Participants then underwent prerandomization assessment with angina severity quantification using Canadian Cardiovascular Society class and Seattle Angina Questionnaire score, cardiopulmonary exercise testing, and dobutamine stress echocardiography. They then attended for the randomization visit, where they had measurement of fasting lipid profile, including low‐density lipoprotein. After being sedated to a deep level of conscious sedation, patients were randomized to PCI or a placebo procedure.

After a 6‐week blinded follow‐up phase, patients returned for repeated symptom assessment, cardiopulmonary exercise testing, and dobutamine stress echocardiography. They were then unblinded and returned to routine care.

Daily home monitoring of pulse and blood pressure was performed throughout using equipment provided by the trial team (Omron M6 monitor; Omron, Kyoto, Japan).

### Medical Therapy

The 6‐week medical optimization phase consisted of telephone consultations 1 to 3 times per week with a consultant cardiologist, supported by regular home blood pressure and heart rate monitoring, to introduce and uptitrate risk reduction and antianginal medication. Medication changes were made by a consultant cardiologist in consultation with study participants. Medication choice was based on national guidelines and individual patient considerations.

**Figure 1:**
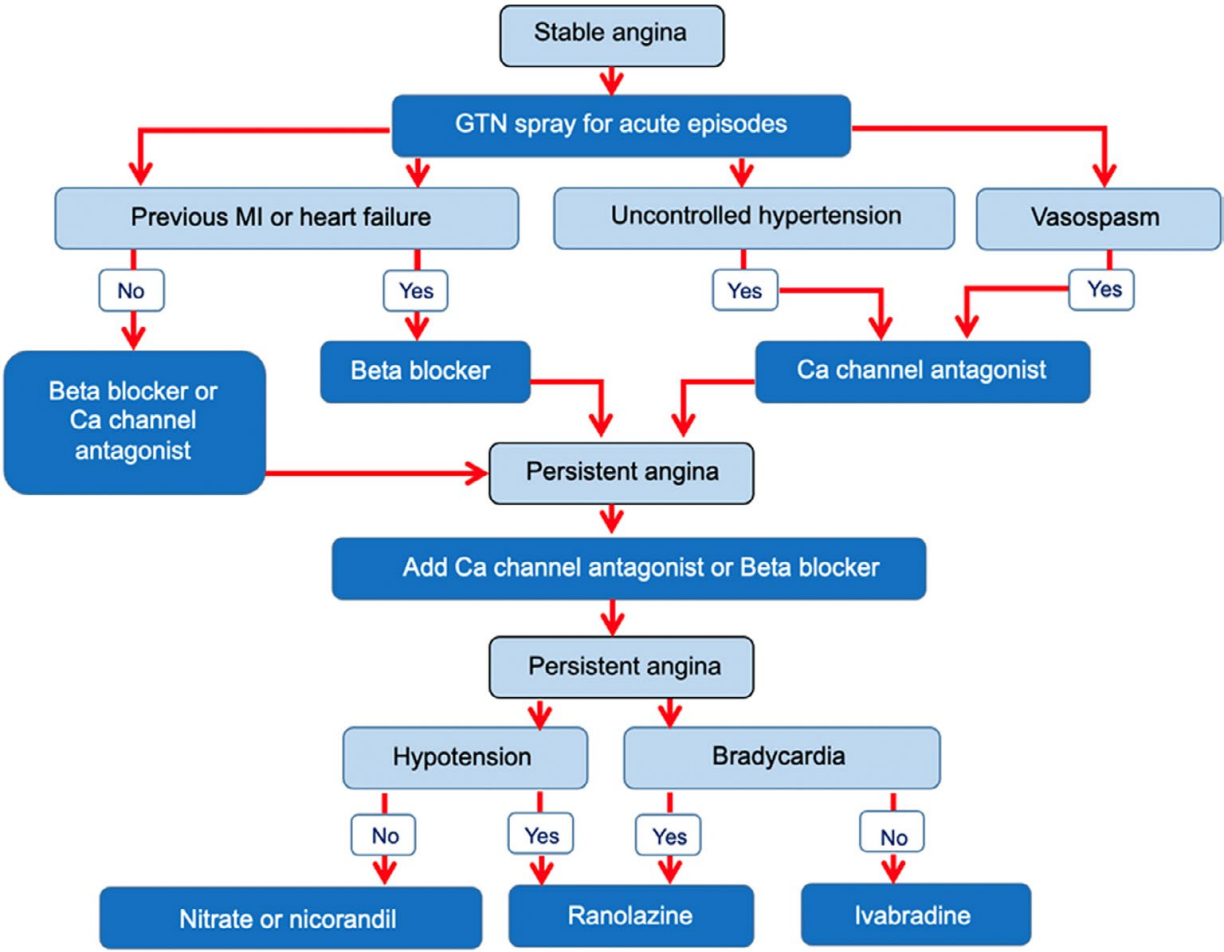
The antianginal decision‐making process that was used in the ORBITA (Objective Randomised Blinded Investigation With Optimal Medical Therapy of Angioplasty in Stable Angina) trial, based on the National Institute of Health and Care Excellence and European Society of Cardiology guidelines.^11^ Ca indicates calcium; GTN, glyceryl trinitrate; and MI, myocardial infarction.

Patients were started on aspirin and a statin, aiming to achieve a dose equivalent to 40 mg atorvastatin. Antianginal therapy was guideline directed, aiming to establish study participants on ≥2 of the following antianginal drugs (or equivalent): bisoprolol, ≥5 mg once a day; amlodipine, ≥5 mg once a day; isosorbide mononitrate, 25 mg once a day; nicorandil, 10 mg twice a day; ivabradine, 7.5 mg twice a day; and ranolazine, 500 mg twice a day (Table 1). The antianginal escalation strategy is shown in Figure 1.^11^ A second antiplatelet agent was started before the randomization procedure.

**Table 1:** Target Doses for Medical Therapy in the ORBITA Trial

Antianginal Therapy (≥2 Antianginals From the Following)	Dose
Bisoprolol (or equivalent β blocker)	≥5 mg once per day
Amlodipine (or equivalent calcium channel antagonist)	≥5 mg once per day
Isosorbide mononitrate (or equivalent long‐acting oral nitrate)	25 mg once per day
Nicorandil	10 mg twice per day
Ivabradine	7.5 mg twice per day
Ranolazine	500 mg twice per day

Risk reduction therapy	Dose
Aspirin	75 mg once per day
Clopidogrel (or equivalent antiplatelet therapy)	75 mg once per day
Atorvastatin	≥40 mg once per day

ORBITA indicates Objective Randomised Blinded Investigation With Optimal Medical Therapy of Angioplasty in Stable Angina.

The ORBITA trial was not designed to detect differences in cardiovascular events. However, to ensure patients received optimal risk reduction therapy, targets of a low‐density lipoprotein cholesterol of <1.8 mmol/L and a systolic blood pressure of <140 mm Hg were used. These were assessed at prerandomization.

An online case reporting form was completed by the study team, which detailed medication initiation, adverse effects, and reasons for medication change and cessation. Most patients were prescribed short‐acting nitrates; these were used at the patient’s discretion. Use of short‐acting nitrates was not recorded.

### Statistical Analysis

Normally distributed data are presented as mean and SD. Nonnormally distributed data are presented as median and interquartile range. The association between the number of antianginal medications used and placebo‐controlled efficacy of PCI was assessed using regression modeling. Models included the prerandomization end point value, the number of antianginal medications tolerated, and the randomization arm. The number of antianginal medications was allowed to interact with the randomization arm. Restricted cubic splines (with 3 knots) were placed on the number of antianginal medications and prerandomization exercise time. Least square models were used for exercise time, proportional odds model was used for Canadian Cardiovascular Society class and Seattle Angina Questionnaire angina frequency, and logistic model was used for freedom from angina. Analyses were performed using the open‐source statistical environment R (version 4.0.2) with the “rms” regression modeling package.^12^

## Results

Medication use and adverse effect data were available for all 200 ORBITA trial patients. Of the original 230 participants who entered the medical optimization phase, 30 left the study before randomization; 5 of these patients left because of medication adverse effects.

### Antianginal Therapy

**Figure 2:**
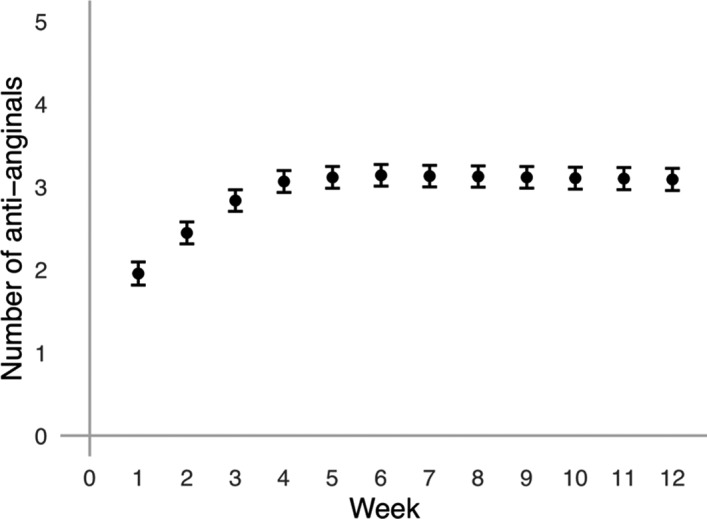
Antianginal drugs per patient from enrollment to follow‐up. Points shown are mean and 95% CI.

The median number of antianginals started (or continued after enrollment) was 4 (interquartile range, 3–4). The median number of antianginals established (taken without stopping the drug for any reason) for each patient was 3 (interquartile range, 2–4). A total of 146 (73.0%) patients were established on ≥3 antianginals; 195 (97.5%) patients reached the trial target of ≥2 antianginals (Table 2). The mean antianginal drugs taken by the end of each week is shown in Figure 2. The percentage of patients on 0 to 5 antianginal drugs by the end of each week is shown in Figure S1.

**Table 2:** Summary of Antianginal Therapy in the ORBITA Trial

Antianginal Therapy	Median (IQR)
Antianginals started or continued at enrollment	4 (3–4)
Antianginals successfully established	3 (2–4)
Antianginals stopped	0 (0–1)

IQR indicates interquartile range; and ORBITA, Objective Randomised Blinded Investigation With Optimal Medical Therapy of Angioplasty in Stable Angina

The median number of antianginals stopped per patient was 0 (interquartile range, 0–1). A total of 136 patients (68.0%) did not stop any antianginals because of adverse effects, 44 (22.0%) patients stopped 1, 19 (9.5%) patients stopped 2, and 1 (0.5%) patient stopped 3.

Fourteen different antianginal drugs were taken by patients in the ORBITA trial. Where <15 patients took a drug, use and adverse effects are not individually reported. Table 3 shows individual antianginal drug use and adverse effect data. Most patients were able to tolerate a high level of antianginal therapy. Calcium channel antagonists were started in 198 (99.0%) patients and were still being taken at follow‐up in 182 (91.9%). Of the 175 (87.5%) patients started on amlodipine, 133 (76.0%) reached the target dose and 4 (2.3%) stopped because of adverse effects. β Blockers were started in 183 (91.5%) patients and were still being taken at follow‐up in 164 (89.6%). Of the 167 patients started on bisoprolol, 91 (54.5%) reached the target dose and 9 (5.4%) stopped for adverse effects.

**Table 3:** Antianginal Therapy in the ORBITA Trial

Drug	Started on Drug (% of Whole Cohort [n=200])	Reached Target Dose (% of Started)	Drug Stopped (% of Started)	Drug Stopped for Adverse Effects (% of Started)	Adverse Effects	On Drug at Follow‐Up (% of Started by Randomization)
Amlodipine	175 (87.5)	133 (76.0)	6 (3.4)	4 (2.3)	Fatigue (1) Dizziness/postural symptoms (2) Ankle edema (1)	170 (97.1)
All calcium channel antagonists	198 (99.0)	142 (71.7)	14 (7.1)	6 (3.0)		182 (91.9)
Bisoprolol	167 (83.5)	91 (54.5)	10 (6.0)	9 (5.4)	Dizziness/postural symptoms (4) Fatigue (3) Blurred vision (1) Insomnia (1)	157 (94.0)
All β blockers	183 (91.5)	98 (53.6)	18 (9.8)	9 (4.9)		164 (89.6)
Isosorbide mononitrate	172 (86.0)	127 (73.8)	37 (21.5)	36 (20.9)	Headache (33) Dizziness/postural symptoms (2) Fatigue (1)	133 (77.3)
Nicorandil	141 (70.5)	102 (72.3)	33 (23.4)	32 (22.7)	Headache (29) Dry eyes (1) Giddiness and nausea (1) Shortness of breath (1)	106 (75.2)
Ranolazine	20 (10.0)	18 (90.0)	1 (5.0)	1 (5.0)	Bruising (1)	19 (95.0)
Ivabradine	18 (9.0)	8 (44.4)	1 (5.6)	1 (5.6)	Cough and shortness of breath (1)	21 (116.7)

ORBITA indicates Objective Randomised Blinded Investigation With Optimal Medical Therapy of Angioplasty in Stable Angina.

Isosorbide mononitrate was started in 172 (86.0%) patients, 127 (73.8%) reached the target dose, and 36 (20.9%) stopped for adverse effects; isosorbide mononitrate was still being taken at follow‐up in 133 (77.3%) of patients. Nicorandil was started in 141 (70.5%) patients, 102 (72.3%) reached the target dose, and 32 (22.7%) stopped for adverse effects; nicorandil was still being taken at follow‐up in 106 (75.2%) of patients. The most common adverse effect experienced by patients taking isosorbide mononitrate and nicorandil was headache.

Ivabradine was started in 18 (9.0%) patients, 8 (44.4%) reached the target dose, and 1 (5.6%) stopped for adverse effects. Three additional patients were started on ivabradine during the follow‐up phase. Ranolazine was started in 20 (10.0%) patients, 18 (90.0%) reached the target dose, and 1 (5.0%) stopped for adverse effects; ranolazine was still being taken at follow‐up in 19 (95%) of patients.

**Figure 3:**
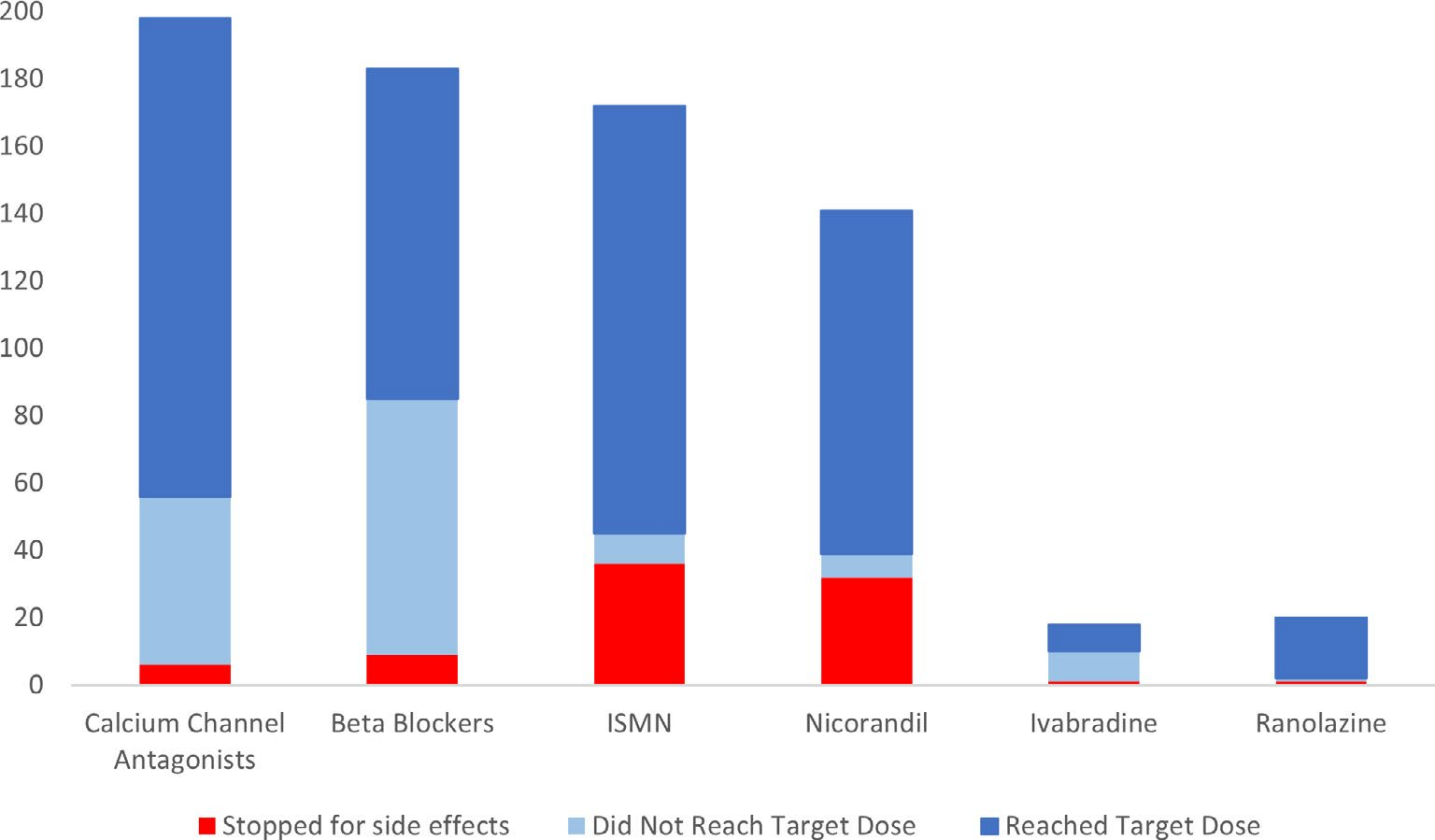
Antianginal medication: number of patients established on each medication class, achieving target dose and stopping for adverse effects. ISMN indicates isosorbide mononitrate.

Figure 3 shows the proportion of patients for each drug class started on therapy, reaching the target dose, and stopping for adverse effects. At follow‐up, the medication use for all drugs and classes was similar to the prerandomization time point.

**Figure 4:**
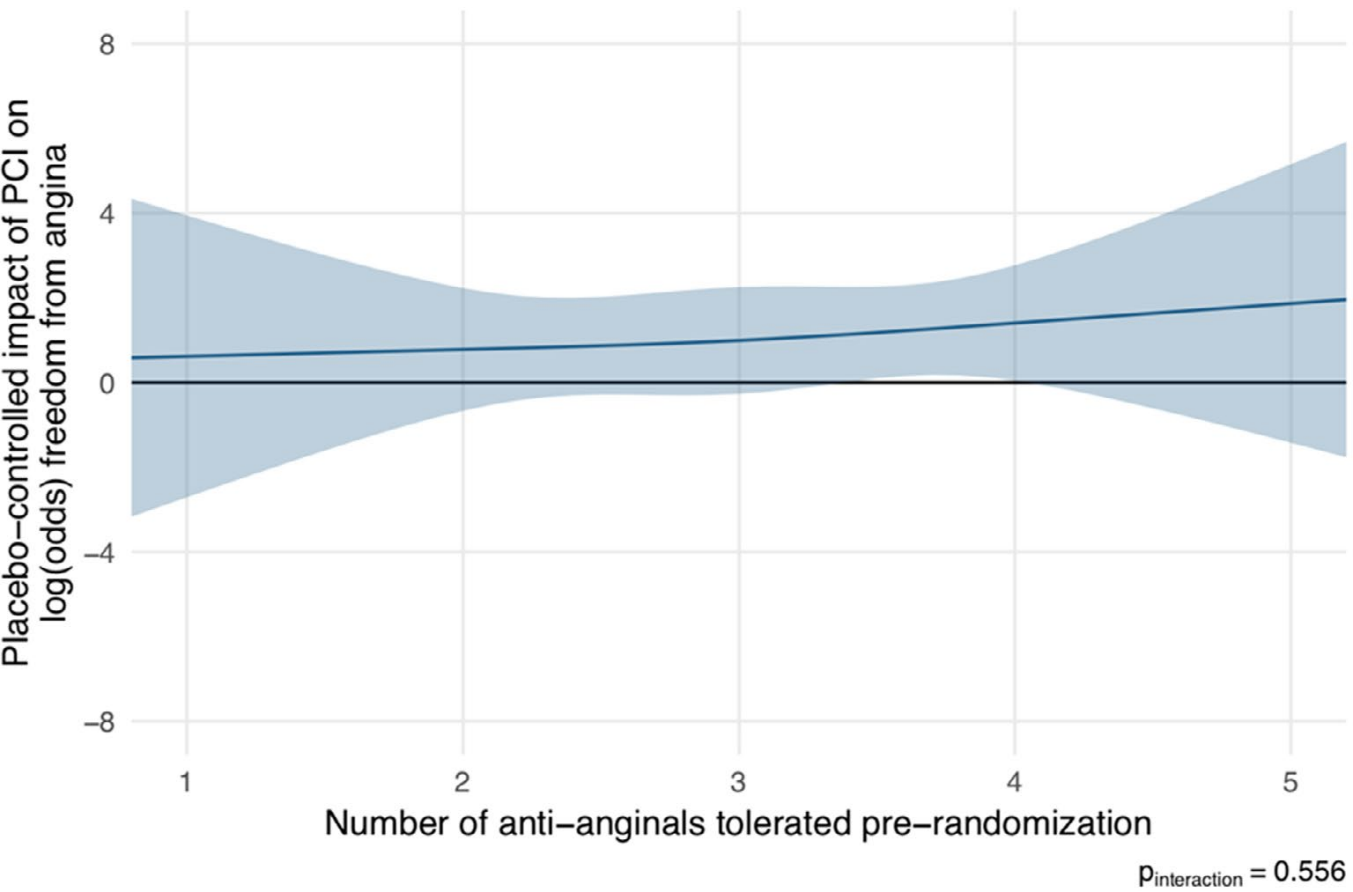
Logistic regression showing the association between the number of prescribed antianginal therapies and log odds of freedom from angina. There is no discernible relationship. PCI indicates percutaneous coronary intervention.

The number of antianginal agents was not associated with the placebo‐controlled efficacy of PCI on freedom from angina (*P*=0.556; Figure 4 *)*. There was similarly no association with change in exercise time (*P*=0.251), Canadian Cardiovascular Society class (*P*=0.765), or Seattle Angina Questionnaire angina frequency (*P*=0.333) (Figures S2–S4). The full logistic regression output is included in the supplemental material, along with Canadian Cardiovascular Society class and Seattle Angina Questionnaire angina frequency at enrollment, prerandomization, and follow‐up (Tables S1–S3).

### Statin and Dual‐Antiplatelet Therapy

Table 4 shows statin therapy. A total of 196 (98.0%) patients were started on at least 1 statin agent; the remaining 4 (2.0%) had previous documented intolerance. At randomization, 191 (95.5%) patients were taking a statin: 174 (87.0%) on atorvastatin, 16 (8%) on rosuvastatin, and 1 (0.5%) on simvastatin. Of the 196 patients started on a statin, 8 (4.1%) experienced adverse effects leading to change of agent or statin cessation. Of the 181 patients started on atorvastatin, 171 (94.5%) achieved the target dose and 4 (2.2%) stopped because of adverse effects. The most common adverse effect was muscle pain (n=3 [1.7%]).

**Table 4:** Statin Therapy in the ORBITA Trial

Drug	Started on Drug (% of Whole Cohort [n=200])	Reached Target Dose (% of Started)	Drug Stopped (% of Started)	Drug Stopped for Adverse Effects (% of Started)	Adverse Effects	On Drug at Follow‐Up (% of Started)
All statins	196 (98.0)	171 (87.2)	5 (2.6)*	4 (2.0)*	Insomnia (1) Stomach pain (1) Foot pain (1) Fatigue (1)	192 (98.0)
Atorvastatin	181 (90.5)	171 (94.5)	7 (3.9)	4 (2.2)	Muscle pain (3) Fatigue (1)	172 (95.0)
Rosuvastatin	19 (9.5)	0 (0.0)	3 (15.8)	3 (15.8)	Insomnia (1) Stomach pain (1) Foot pain (1)	18 (94.7)

ORBITA indicates Objective Randomised Blinded Investigation With Optimal Medical Therapy of Angioplasty in Stable Angina.

* These numbers are lower than individual statin agent drug cessations as some patients who stopped one statin were successfully started on another. Five patients stopped one statin and were not able to tolerate another. Four stopped for adverse effects and did not successfully start another. Eight patients stopped for adverse effects (4 on atorvastatin, 3 on rosuvastatin, and 1 on simvastatin).

All 200 patients were started on at least one antiplatelet agent, with 194 (97.0%) on dual‐antiplatelet therapy. Adverse effects leading to antiplatelet cessation were uncommon, with only 1 patient temporarily stopping aspirin for upper gastrointestinal bleeding and 1 stopping ticagrelor for shortness of breath. The antiplatelet and statin data are shown in Figure S5.

At prerandomization, mean low‐density lipoprotein was 1.84±0.74 mmol/L, with 102 (51.0%) patients reaching target. Mean systolic blood pressure was 125.6±16.9 mm Hg, with 159 (79.5%) patients reaching target. The blood pressure and heart rate at enrollment, prerandomization, and follow‐up are reproduced from a previous publication in Table S4.^10^

## Discussion

In this analysis of medical therapy in the ORBITA trial, we have shown that, within a 12‐week trial period, it is possible to achieve guideline‐directed truly optimal medical therapy. Antianginal therapy was initiated and uptitrated to effective doses in the majority. Amlodipine, bisoprolol, ranolazine, and ivabradine were well tolerated, whereas isosorbide mononitrate and nicorandil were associated with more adverse effects than other antianginal agents. Statins were well tolerated, causing adverse effects in only a small minority of patients.

### Antianginal Therapy in Trials of PCI

The first trial of PCI for angina in the balloon angioplasty era, the ACME (Angioplasty Compared to Medicine) trial, used a “stepped‐care approach” with medical therapy in the control arm. Relatively low levels of medical therapy were achieved at 6 months, with 59%, 50%, and 71% of patients taking nitrates, β blockers, and calcium channel antagonists, respectively, in the control arm, and much lower levels in the treatment arm.^13^ Multiple trials in stable CAD followed; however, the importance of attempting to attain truly optimal medical therapy was emphasized and integrated into trial design by the COURAGE (Clinical Outcomes Utilizing Revascularization and Aggressive Drug Evaluation) trial.^3^ At randomization, 67.4%, 86.8%, and 41.4% of patients were on a nitrate, β blocker, and calcium channel antagonist, respectively. Medical therapy was more modest in the subsequent FAME 2 (Fractional Flow Reserve Versus Angiography for Multivessel Evaluation 2) trial, with 76.7% on β blockers and only 23.1% on calcium channel antagonists. Nitrate use was unreported. More recently, the ISCHEMIA trial was designed to compare an initial invasive or conservative strategy, in patients with moderate to severe ischemia. Despite the complexity of medication optimization in this large, multicenter, international trial, a great deal of effort was invested in trying to attain optimal medical therapy.^14^ There was a high level of β‐blocker use, 81.0%, with lower levels of calcium channel antagonist use, 30.2%, at enrollment,^14^ and similar proportions at follow‐up.^5^

This analysis has demonstrated that the average ORBITA trial patient was on a much higher level of antianginal therapy than in routine clinical practice or other trials of PCI.^3^, ^4^, ^5^, ^13^ This may be for a variety of reasons. First, for optimal medical therapy to be achieved, it must be imbedded in the trial design with strict protocols and plans for medication introduction, response evaluation, and changes, where necessary. Second, teams must be well trained and patients must be willing to accept the medication protocol. However, it must be remembered that the length of a clinical trial period may have an impact on persistence and adherence to optimal medical therapy. Higher levels of therapy may be easier to maintain over a shorter clinical trial period, such as the 12 weeks of the ORBITA trial. This must be taken into consideration when comparing clinical trials and considering translation into clinical practice.

The incremental efficacy of PCI in a clinical trial may depend on the level of background medical therapy that is established.^1^ For a subjective end point, such as symptom relief, the effect of any treatment will consist of a combination of both a physical and a placebo component.^15^ All unblinded trials of revascularization versus a conservative approach in stable CAD showed improvement in symptoms of angina.^5^, ^13^, ^16^, ^17^ Some of this treatment effect may have been caused by placebo, but it is also possible that the benefit of PCI is increased if introduced on lower levels of medical therapy. High levels of antianginal therapy in the ORBITA trial may have resulted in a reduction in the potential incremental impact of PCI on the physical component of the treatment effect. Perhaps as a result, PCI only demonstrated minimal placebo‐controlled symptom efficacy on one secondary end point of patient‐reported freedom from angina and has small but nonnegligible short‐ and long‐term adverse effects.^18^

This phenomenon is analogous to the data from trials of renal denervation. In the early unblinded renal denervation trials, with patients on modest levels of antihypertensive therapy, the effect of renal denervation on blood pressure reduction was pronounced.^19^, ^20^ Later, the first placebo‐controlled trial of denervation on a background of intense antihypertensive therapy showed a much smaller and not statistically significant effect size.^21^ More recently, renal denervation has been tested in patients off antihypertensive therapy, showing a larger effect size of the interventional therapy.^22^ These data suggest that the effect size of an intervention can be magnified by introducing the intervention to patients on lower levels of medical therapy, thereby increasing the physical component of its treatment effect.

Alternatively, it may be that if a patient with stable angina has a good response to antianginal medication, we can be certain that the symptoms are related to CAD. If, however, angina does not improve despite introduction of multiple antianginal agents, it may be more likely that the symptoms are not cardiac. Therefore, perhaps patients with the best response to antianginal therapy can be expected to have the greatest symptomatic improvement with revascularization.

Although, in this analysis of the ORBITA trial, there was no association between the number of antianginal medications at prerandomization and the placebo‐controlled efficacy of PCI, this may be because the cohort was on high levels of therapy (97.5% of patients on ≥2 antianginal medications by randomization). Therefore, there may have been insufficient variation in antianginal medication across the trial population to adequately test this possible association.

### Clinical Implications

In clinical practice, patients are often not established on adequate medical therapy before being referred for PCI. In a UK study, only 32% of patients were on 2 antianginal drugs before being referred for PCI, with 9% of patients on no antianginal drugs at all.^8^ This may in part be because of concern about the tolerability of the regimen, polypharmacy, and possible adverse effects. The process of introduction and uptitration of antianginal therapy may be seen as labor intensive and unrealistic in current clinical practice. In addition, patients and physicians may consider an upfront PCI procedure to be simpler, more readily accessible, and preferable. We have demonstrated that, in the context of a 12‐week clinical trial, antianginal therapy is well tolerated in the majority and that adverse effects leading to drug stoppages are rare. In particular, amlodipine and bisoprolol were well tolerated, with the highest rates of adverse effects seen with nicorandil and isosorbide mononitrate. In light of the results of the ISCHEMIA trial, clinicians should be confident to prescribe and uptitrate antianginal therapy in the knowledge they are not denying their patient a procedure with prognostic benefit. However, in clinical practice, patients may not want to take ≥3 antianginal drugs long‐term, preferring to pursue PCI. The ORBITA‐2 trial will further address this question by investigating the placebo‐controlled efficacy of PCI in patients taking real‐world antianginal therapy.^23^

### Study Limitations

Within the ORBITA trial, patient‐physician interaction was more intense than would be realistic in routine clinical practice. With 2 to 3 telephone consultations with a physician per week, it is possible that patients may have been more likely to tolerate higher degrees of medical therapy than would be achievable in routine clinical practice. However, the frequency of interaction was driven by the necessity to complete the medical optimization phase within 6 weeks to ensure that the trial was feasible, ethical, and acceptable. In routine clinical practice, consultations could be distributed over a longer time to achieve the same effect.

This analysis of the ORBITA trial demonstrates the feasibility of rapidly uptitrating antianginal therapy in a clinical study setting with a relatively short duration of follow‐up. Of 230 patients enrolled into the trial, 5 left before randomization because of medication adverse effects. It is possible that the 200 randomized patients may have been more prepared to undergo the intensive study regimen than the wider clinical population. High levels of antianginal drug therapy may be less tolerable over a longer period, with poorer persistence and adherence in longer‐term clinical trials and outside of the setting of clinical research.

Patients and physicians were not blinded to the medications used in the ORBITA trial. This could have influenced the unblinded adverse effect reporting. As a UK‐based clinical trial, within the National Health Service, patients did not need to pay for medication. This will not be the case in all healthcare systems and may limit the international applicability of these results. Also, nicorandil and ivabradine are not licensed as antianginal therapies in the United States.

## Conclusions

The ORBITA trial shows that optimal medical therapy with guideline‐directed antianginals, statins, and dual antiplatelet therapy is achievable and well tolerated by most patients in a 12‐week trial period. With protocolized medication initiation and uptitration, it is possible to achieve truly optimal medical therapy. Future studies are required to assess the efficacy of this protocol in longer‐term clinical practice.

## Sources of Funding

The ORBITA (Objective Randomised Blinded Investigation With Optimal Medical Therapy of Angioplasty in Stable Angina) trial was an investigator‐led trial sponsored by Imperial College London. We acknowledge the support of the National Institute for Health Research (NIHR) Clinical Research Network, NIHR Imperial Biomedical Research Centre, Foundation for Circulatory Health, Imperial College Healthcare Charity, Philips Volcano, and The British Heart Foundation Centre of Research Excellence at Imperial.

## Disclosures

Dr Sharp is a consultant for Philips Volcano. Drs Sen, Petraco, and Nijjer have received speaker’s honoraria from Philips Volcano. Dr Al‐Lamee has received speaker’s honoraria from Philips Volcano and Menarini Pharmaceuticals. Dr Keeble has received research grants from Philips Volcano. The remaining authors have no disclosures to report.

## Supporting information


**Tables S1–S4**
**Figures S1–S5**
