# Supplementary material for: Achieving Optimal Medical Therapy: Insights From the ORBITA Trial
Source: J Am Heart Assoc. 2021 Jan 26;10(3):e017381. doi: 10.1161/JAHA.120.017381 (PMC7955412; doi:10.1161/JAHA.120.017381)

Supplemental Material

Table S1. Logistic (Proportional Odds) Ordinal Regression Model.
orm(formula = outcome_anginafree_post ~ outcome_anginafree_pre + Number_AA_Tolerated * pci_or_placebo, data = d_pr)

	**Model Likelihood Ratio Test**	**Discrimination Indexes**	**Rank Discrim. Indexes**
Obs 192	LR χ^2 27.78	R^2 0.182	ρ 0.376
FALSE 114	d.f. 4	*g* 0.914	
TRUE 78	Pr(>χ^2) <0.0001	g_r 2.493	
Distinct *Y* 2	Score χ^2 26.80	|Pr(*Y* ≥ median)-½| 0.177	
$Y_{0.5}$ 1	Pr(>χ^2) <0.0001		
max |∂log *L*/∂β| 0.0001			

	**β**	**S.E.**	**Wald *Z***	**Pr(>|*Z*|)**
Intercept	-1.1120	0.6564	-1.69	0.0902
outcome_anginafree_pre	1.8191	0.4459	4.08	<0.0001
Number_AA_Tolerated	0.2864	0.2108	1.36	0.1742
pci_or_placebo=Placebo	-0.3281	1.1950	-0.27	0.7837
Number_AA_Tolerated × pci_or_placebo=Placebo	-0.2176	0.3693	-0.59	0.5558

Wald Statistics for outcome_anginafree_post			
	χ^2	**d.f.**	***P***
outcome_anginafree_pre	16.65	1	<0.0001
Number_AA_Tolerated (Factor+Higher Order Factors)	1.90	2	0.3871
All Interactions	0.35	1	0.5558
pci_or_placebo (Factor+Higher Order Factors)	9.57	2	0.0083
All Interactions	0.35	1	0.5558
Number_AA_Tolerated × pci_or_placebo (Factor+Higher Order Factors)	0.35	1	0.5558
TOTAL	22.87	4	0.0001

Table S2. Physician Assessed Severity of Angina.

		PCI n(%)	Placebo n(%)
Enrolment	Class	n=105	n=95
CCS	0	0 (0)	0 (0)
	1	2 (1.9)	3 (3.2)
	2	64 (61.0)	54 (56.8)
	3	39 (37.1)	38 (40.0)
Pre-randomization		n=105	n=95
CCS	0	9 (8.6)	13 (13.7)
	1	15 (14.3)	10 (10.5)
	2	56 (53.3)	41 (43.2)
	3	25 (23.8)	31 (32.6)
Follow up		n=105	n=91
CCS	0	41 (39.0)	26 (28.6)
	1	14 (13.3)	18 (19.8)
	2	37 (35.2)	31 (34.1)
	3	13 (12.4)	15 (16.5)
	4	0 (0)	1 (1.1)

CCS = Canadian Cardiovascular Society
PCI = percutaneous coronary intervention

Table S3. Seattle Angina Questionnaire Frequency of Angina.

		PCI n(%)	Placebo n(%)
Enrolment		n=103	n=92
Frequency of angina	>4 x /day	3 (2.9)	7 (7.6)
	1-3 x /day	29 (28.2)	20 (21.7)
	≥3x / week but not every day	23 (22.3)	21 (22.8)
	1-2 x / week	17 (16.5)	13 (14.1)
	<1x /week	18 (17.5)	17 (18.5)
	None in the past 4 weeks	13 (12.6)	14 (15.2)
Pre-randomization		n=104	n=94
Frequency of angina	>4 x /day	2 (1.9)	4 (4.3)
	1-3 x /day	19 (18.3)	18 (19.2)
	≥3x / week but not every day	15 (14.4)	17 (18.1)
	1-2 x / week	25 (24.0)	18 (19.1)
	<1x /week	28 (26.9)	18 (19.1)
	None in the past 4 weeks	15 (14.4)	19 (20.2)
Follow up		n=103	n=91
Frequency of angina	>4 x /day	2 (1.9)	4 (4.4)
	1-3 x /day	7 (6.8)	8 (8.8)
	≥3x / week but not every day	13 (12.6)	12 (13.2)
	1-2 x / week	14 (13.6)	21 (23.1)
	<1x /week	16 (15.5)	18 (19.8)
	None in the past 4 weeks	51 (49.5)	28 (30.8)

PCI = percutaneous coronary intervention
These were the answers provided by the patients to the following written question from the Seattle Angina Questionnaire: “Over the past 4 weeks, how many times have you had chest pain, chest tightness or angina?”

Table S4. The blood pressure and heart rate data from the ORBITA trial, at enrolment, randomisation and follow up, by randomisation arm.

	Systolic BP (mmHg)		Diastolic BP (mmHg)		Heart rate (bpm)	
	**PCI**	**Placebo**	**PCI**	**Placebo**	**PCI**	**Placebo**
Enrolment	136·5 (16·9) n=105	137·9 (18·6) n=95	76·6 (11·6) n=105	76·2 (9·6) n=95	65·3 (13·0) n=105	66·2 (11·3) n=95
Pre-randomisation	125·3 (16·9) n=105	126·0 (17·0) n=95	70·4 (11·1) n=105	70·4 (11·0) n=95	60·5 (11·1) n=105	61·9 (9·2) n=95
Follow-up	130·5 (17·5) n=105	133·4 (20·0) n=91	70·8 (11·8) n=105	74·0 (12·7) n=91	62·7 (11·1) n=105	66·2 (13·5) n=91
Δ (Enrolment to pre-randomisation)	-11·5 (18·0) **(p<0·001)**	-11·9 (21·1) **(p<0·001)**	-6·3 (10·8) **(p<0·001)**	-5.8 (12·5) **(p<0·001)**	-4·7 (11·3) **(p<0·001)**	-4·3 (12·3) **(p<0·001)**
Difference in Δ between groups (Enrolment to pre-randomisation)	0·4 (95% CI -5·1 to 5·9) (p=0·88)		-0·6 (95% CI -3·8 to 2·7) (p=0·74)		-0·4 (95% CI -3·7 to 2·9) (p=0·81)	
Δ (Pre-randomisation to follow-up)	5·3 (18·0) **(p=0·004)**	7·3 (18·6) **(p=0·004)**	0·5 (12·0) (p=0·65)	3·3 (12·5) **(p=0·01)**	2·1 (10·4) **(p=0·04)**	4·2 (12·8) **(p=0·002)**
Difference in Δ between groups (Pre-randomisation to follow-up)	-2·0 (95% CI -7·2 to 3·2) (p=0·44)		-2·7 (95% CI -6·2 to 0·8) (p=0·12)		-2·1 (95% CI -5·4 to 1·2) (p=0·20)	

Data are mean (SD). PCI=percutaneous coronary intervention. BP= blood pressure.

Figure S1. The percentage of patients on 0 to 5 antianginal drugs each week from enrolment to follow up.

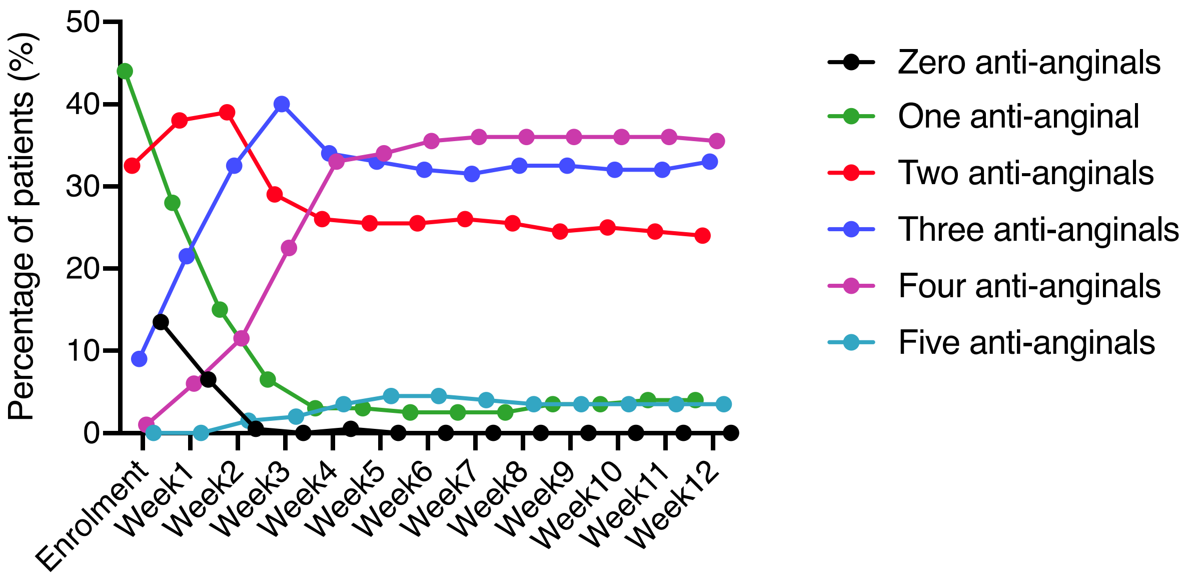

Figure S2. Regression analysis, showing no impact of the number of prescribed anti-anginal therapies on the log odds of improvement in CCS class post randomization to PCI or placebo.

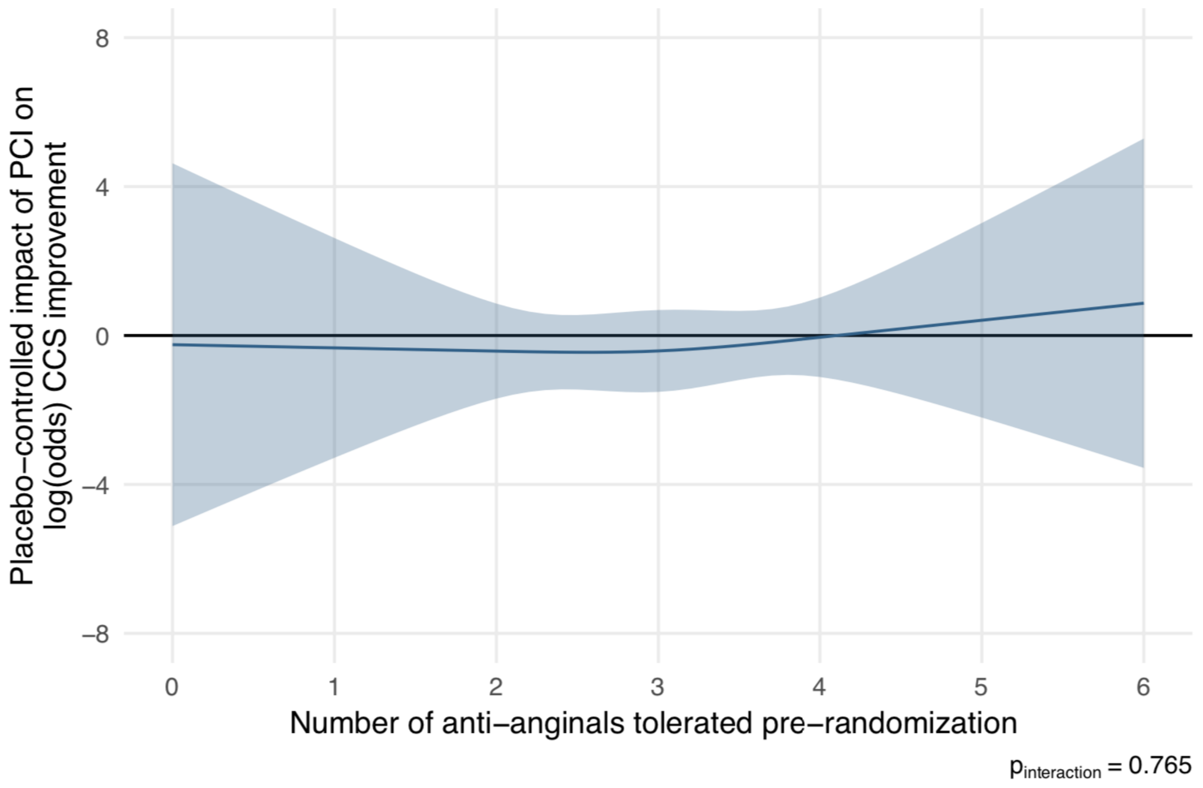

Figure S3. Regression analysis, showing no impact of the number of prescribed anti-anginal therapies on exercise time post randomization to PCI or placebo.

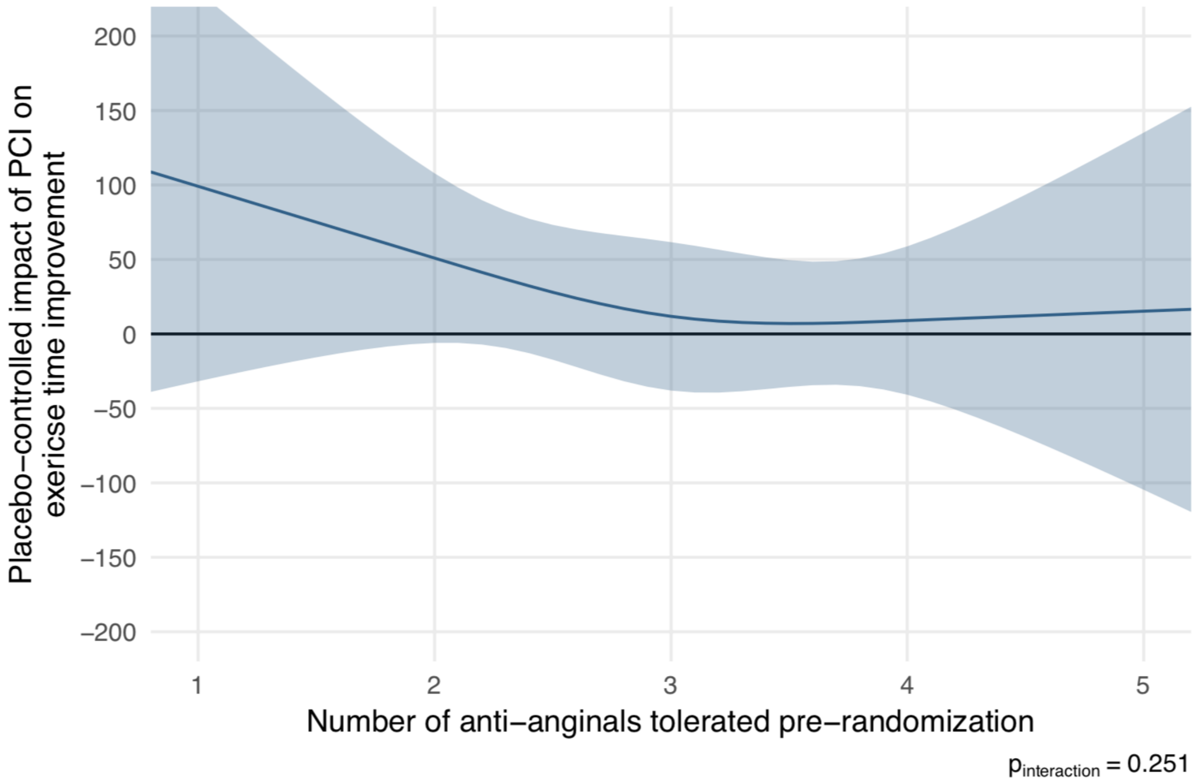

Figure S4. Regression analysis, showing no impact of the number of prescribed anti-anginal therapies on log odds SAQ angina frequency post randomization to PCI or placebo.

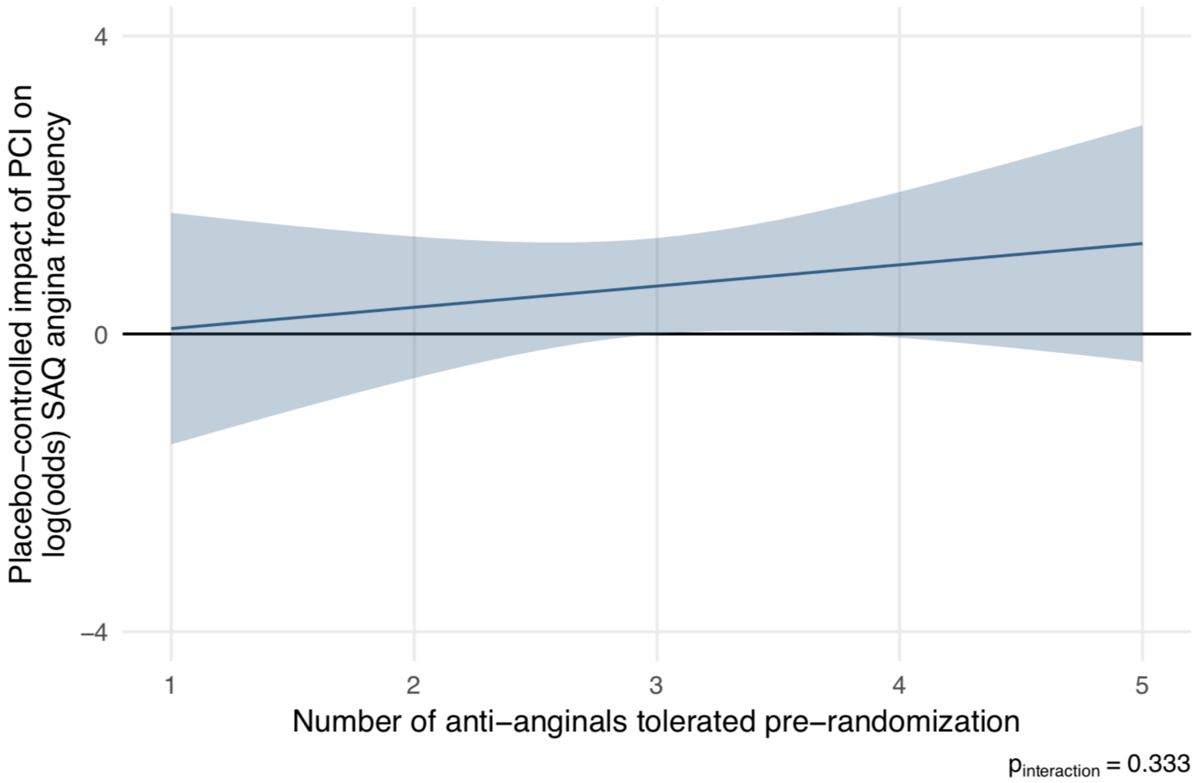

Figure S5. The anti-platelet and statin therapy in the ORBITA trial.

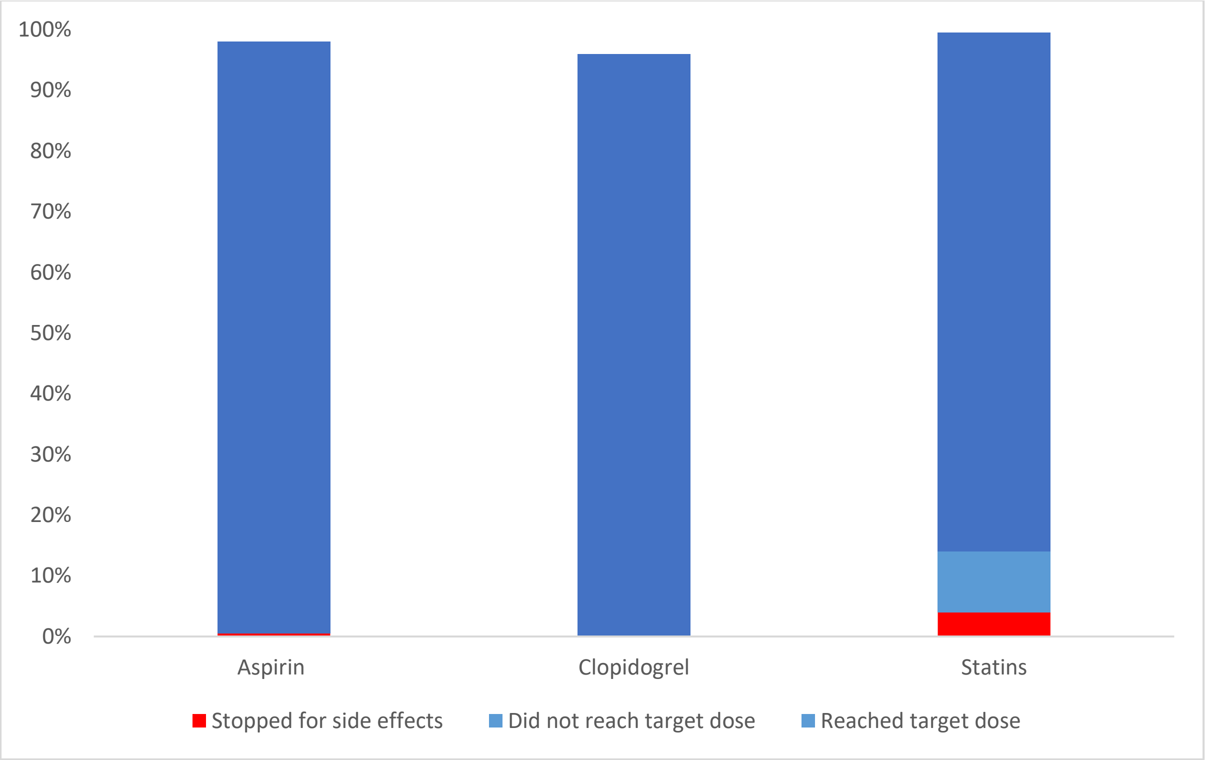

Supplement: Supplementary file 1 — Tables S1–S4 Figures S1–S5 [file JAH3-10-e017381-s001.pdf]
